# A Comprehensive Review of the Role of Biomarkers in the Early Detection of Endocrine Disorders in Critical Illnesses

**DOI:** 10.7759/cureus.61409

**Published:** 2024-05-31

**Authors:** Pallavi Deulkar, Amol Singam, Abhishek Jain

**Affiliations:** 1 Critical Care Medicine, Jawaharlal Nehru Medical College, Datta Meghe Institute of Higher Education and Research, Wardha, IND

**Keywords:** patient outcomes, intensive care unit (icu), early detection, biomarkers, critical illness, endocrine disorders

## Abstract

Endocrine disorders pose significant challenges in the management of critically ill patients, contributing to morbidity and mortality in intensive care settings. Timely detection of these disorders is essential to optimizing patient outcomes. Biomarkers, as measurable indicators of biological processes or disease states, play a crucial role in the early identification and monitoring of endocrine dysfunction. This comprehensive review examines the role of biomarkers in the early detection of endocrine disorders in critical illnesses. We provide an overview of common endocrine disorders encountered in the intensive care unit (ICU) and discuss the impact of endocrine dysregulation on patient outcomes. Additionally, we classify biomarkers and explore their significance in diagnosing and monitoring endocrine disorders, including thyroid dysfunction, adrenal insufficiency, and hypopituitarism. Furthermore, we discuss the clinical applications of biomarkers, including their utility in guiding therapeutic interventions, monitoring disease progression, and predicting outcomes in critical illnesses. Emerging trends and future directions in biomarker research are also highlighted, emphasizing the need for continued investigation into novel biomarkers and technological advancements. Finally, we underscore the potential of biomarkers to revolutionize the early detection and management of endocrine disorders in critical illnesses, ultimately improving patient care and outcomes in the ICU.

## Introduction and background

Endocrine disorders encompass a broad spectrum of conditions characterized by dysfunction in the endocrine system, which comprises glands that produce hormones regulating various bodily functions such as metabolism, growth, and reproduction. These disorders can arise from hormone overproduction, underproduction, or impaired hormone action [1]. In critical illness, timely identification of endocrine disorders is paramount due to their potential to exacerbate morbidity and mortality. Endocrine dysregulation can significantly impact vital physiological processes, including cardiovascular function, immune response, and glucose metabolism, thereby complicating the management of critically ill patients [2].

Biomarkers, measurable indicators of biological processes or disease states, play a crucial role in the early detection, diagnosis, and monitoring of endocrine disorders in critical illnesses. These biomarkers encompass a wide range of molecules, including hormones, enzymes, proteins, and genetic markers, which can provide valuable insights into the pathophysiological mechanisms underlying endocrine dysfunction [3]. This review aims to comprehensively evaluate the role of biomarkers in the early detection of endocrine disorders in critical illnesses. By synthesizing current evidence and identifying emerging trends, this review seeks to elucidate the diagnostic utility of biomarkers, their clinical applications, and their potential to improve patient outcomes in the critical care setting.

## Review

Endocrine disorders in critical illness

Common Endocrine Disorders Encountered in Critical Care Settings

**Figure 1:**
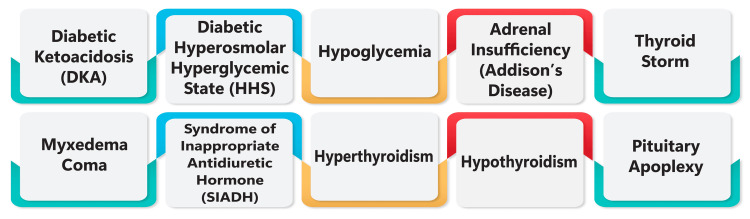
Common endocrine disorders encountered in critical care settings The image is created by the corresponding author.

Common endocrine disorders encountered in critical care settings encompass non-insulin-dependent diabetes mellitus, obesity, malnutrition, and chronic heart failure, all of which represent prevalent comorbidities among critically ill patients [4]. Moreover, endocrine emergencies, such as adrenal insufficiency, thyroid crisis, hypoglycemia, hyperglycemic crisis, and electrolyte abnormalities like hypercalcemia and hypocalcemia, are frequently observed in critically ill patients and can pose life-threatening risks if not promptly identified and addressed [5-7]. These emergent endocrine conditions often manifest with nonspecific symptoms that can mimic other medical conditions, underscoring the importance of maintaining a vigilant approach in critical care settings [6]. Prompt and targeted management of endocrine emergencies is paramount to enhancing patient outcomes and mitigating mortality rates in the intensive care unit [6]. Figure 1 shows common endocrine disorders encountered in critical care settings.

Impact of Endocrine Dysregulation on Patient Outcomes

Endocrine-disrupting chemicals (EDCs) pose significant threats to human health, manifesting in a multitude of adverse effects such as compromised sperm quality and fertility, anomalies in sexual organs, endometriosis, precocious puberty, and metabolic disturbances like diabetes and obesity [8]. These chemicals disrupt hormonal equilibrium by mimicking, obstructing, or altering the production and function of various hormones [8]. In critically ill patients, the dynamic nature of the endocrine system can obscure pre-existing endocrine disorders, complicating their identification [9]. Critical illness triggers profound endocrine alterations uncommon in non-critical scenarios, prompting inquiries into the necessity and efficacy of endocrine interventions to enhance patient outcomes [9]. Endocrine emergencies, such as adrenal crises and thyroid storms, are prevalent among critically ill individuals, necessitating swift recognition and treatment for lifesaving intervention [10]. The findings underscore the profound repercussions of endocrine dysregulation, whether stemming from environmental exposures or critical illness, on patient well-being and clinical prognoses. Rigorous assessment and management of endocrine irregularities are imperative, particularly in the intensive care milieu where endocrine dysfunction is prevalent and can substantially impact patient trajectories [10].

Challenges in Timely Diagnosis and Management

The challenges in achieving timely diagnosis and management of health issues encompass a spectrum of factors that impede prompt identification and treatment. These hurdles include delays in seeking medical attention, limited accessibility to healthcare services, low awareness of symptoms, and limited resources for diagnostic testing [11, 12]. Amid the backdrop of the COVID-19 pandemic, the redirection of resources toward combating the virus has resulted in diminished manpower for routine and diagnostic tests, culminating in delays in cancer diagnosis and management [13]. Moreover, apprehensions surrounding contracting the illness and ensuring workplace safety have further complicated the expeditious diagnosis and treatment of cancer patients during this period [13]. Furthermore, disruptions in cancer healthcare services due to compromised facilities, supply chain disruptions, and shortages in personnel availability have profoundly impacted the treatment, diagnosis, and overall health service provision for cancer patients [13]. Addressing these challenges necessitates substantial investments in healthcare infrastructure, research, and development, as well as comprehensive training and education programs for healthcare professionals. Additionally, the implementation of innovative solutions such as telemedicine and mobile testing vans can play a pivotal role in ensuring timely diagnosis and management of health conditions [12, 13].

Biomarkers as diagnostic tools

Definition and Classification of Biomarkers

A biomarker represents a molecular, cellular, or biochemical alteration that can be consistently and accurately measured, serving as an indicator of normal biological processes, pathological changes, or responses to therapeutic interventions. These biological indicators are instrumental in disease research, facilitating disease diagnosis, monitoring treatment efficacy, and advancing drug development. Biomarkers encompass a broad spectrum of biomolecules, including carbohydrates, proteins, lipids, genes, DNA, RNA, platelets, enzymes, and hormones, among others. They play a pivotal role in disease identification, guiding treatment decisions, and evaluating disease progression and treatment outcomes. Biomarkers can be detected using various methodologies, such as blood tests and imaging modalities like MRI, thereby enhancing their applicability in clinical settings [14,15]. The classification of biomarkers is based on their functions and roles in disease identification, progression, and clinical management. These classifications include prognostic, predictive, pharmacodynamic, and exposure-related biomarkers, each serving a distinct purpose in understanding and managing various diseases. Prognostic biomarkers forecast the likelihood of future clinical events, such as disease recurrence or progression, in patients with established conditions. Predictive biomarkers indicate the probability of a patient's response to a specific therapeutic intervention. Pharmacodynamic biomarkers aid in elucidating the effects of medications on physiological processes, while exposure-related biomarkers offer insights into the body's response to external factors such as medications or environmental exposures. Additionally, biomarkers may belong to multiple classification types, underscoring their multifaceted roles in disease diagnosis and treatment monitoring [14,15].

Role of Biomarkers in Early Detection of Endocrine Disorders

Biomarkers play a pivotal role in the early detection of endocrine disorders, facilitating timely diagnosis and effective treatment monitoring. These measurable indicators of hormonal imbalances or abnormalities provide crucial insights into an individual's physiological processes, cellular changes, and biochemical imbalances, thereby aiding in disease detection, risk assessment, and treatment monitoring [16]. In the realm of endocrinology, biomarkers are instrumental in assessing endocrine system function, diagnosing disorders, monitoring disease progression, and evaluating treatment efficacy [16]. In the context of endocrine neoplasms, biomarkers are utilized in conjunction with biochemical, imaging, and genetic analyses to accurately characterize tumors. Common serum biomarkers such as chromogranin A (CgA), cancer antigen (CA), beta-human chorionic gonadotropin (hCG), and alpha-fetoprotein (AFP) are employed across various endocrine tumors to aid in diagnosis and management. Moreover, ongoing research is exploring the potential of microRNAs (miRNAs) as novel biomarkers for different tumor types, thereby enhancing our understanding and diagnostic capabilities in the field of endocrine disorders [17]. Biomarkers in endocrinology encompass a diverse array of types, including genomic biomarkers, proteomic biomarkers, metabolomics, and applications of artificial intelligence. These biomarkers are pivotal in early disease detection, prognosis, and treatment monitoring, empowering healthcare professionals to deliver more tailored and effective care to patients with endocrine-related conditions. Ultimately, the integration of biomarkers into clinical practice holds the promise of improving patient outcomes and advancing personalized medicine within the field of endocrinology [17].

Advantages and Limitations of Biomarker-Based Diagnostics

Biomarkers serve as invaluable early indicators of disease, enabling earlier diagnosis and intervention [18,19]. They offer objective and quantitative measurements that aid in disease detection, monitoring, and prognosis [18,19]. Additionally, biomarkers facilitate patient stratification and guide the development of personalized treatment approaches [18,19]. Technological advancements, particularly in fields like proteomics and metabolomics, have broadened the repertoire of novel biomarkers available for various diseases [19]. Moreover, combining multiple biomarkers has been shown to enhance diagnostic accuracy compared to relying on a single biomarker [19]. However, identifying biomarkers with ideal sensitivity and specificity presents a significant challenge [20]. Biomarker research often suffers from shortcomings in selection, standardization, and validation, thereby limiting their clinical translation [20]. Additionally, technical limitations in current biomarker detection methods, such as restricted sensitivity and dynamic range, can impede their utility [21]. The interpretation of biomarker results is further complicated by factors such as patient characteristics and sample handling, which can influence measurements [20]. Moreover, the regulatory approval and integration of new biomarkers into clinical practice entail a lengthy and costly process [20]. Furthermore, the lack of standardization and harmonization across different research laboratories and clinical trials poses challenges in comparing biomarker performance [20].

Specific biomarkers for endocrine disorders

Thyroid Dysfunction

Thyroid hormones, including triiodothyronine (T3) and thyroxine (T4), are fundamental hormones synthesized by the thyroid gland. Alterations in these hormones serve as indicators of thyroid dysfunction [22]. Thyroid-stimulating hormone (TSH) holds pivotal importance as a biomarker for thyroid function. Elevated levels of TSH typically signify hyperthyroidism, while reduced levels suggest hypothyroidism [22,23]. Thyroglobulin (Tg), a protein synthesized by thyroid follicular cells, serves as a marker for thyroid cancer when its levels are elevated. It also aids in diagnosing and monitoring thyroid dysfunction [24]. Thyroid autoantibodies, such as anti-thyroglobulin antibody (TgAb) and anti-thyroperoxidase antibody (TPOAb), are associated with autoimmune thyroid diseases like Graves' disease and Hashimoto's thyroiditis [23,24]. Certain cytokines and chemokines, like interleukin (IL)-1β, IL-2, IL-8, Granulocyte-macrophage colony-stimulating factor (GM-CSF), granulocyte-colony stimulating factor (G-CSF), and monocyte chemoattractant protein-1 (MCP-1), have been implicated in the onset of thyroid dysfunction during immune checkpoint inhibitor treatment for cancer [25]. These biomarkers play a crucial role in diagnosing thyroid dysfunction, distinguishing between different thyroid disorders, and monitoring treatment responses. However, it's noteworthy that while serum thyroid hormone concentrations within the reference range may appear indicative of euthyroidism, this might not always reflect the status of thyroid hormones in all tissues. Further research is imperative to establish reliable serum markers accurately reflecting tissue thyroid hormone status [22].

Adrenal Insufficiency

Adrenal insufficiency, also recognized as Addison's disease, manifests when the adrenal glands fail to produce adequate levels of steroid hormones, comprising cortisol, aldosterone, and androgens. This insufficiency triggers an array of symptoms, encompassing fatigue, muscle weakness, weight loss, abdominal discomfort, and, in severe cases, life-threatening conditions like organ failure and shock. The condition can originate from primary causes, originating within the adrenal glands themselves, or secondary causes, stemming from dysfunctions in the pituitary gland or hypothalamus that disrupt hormone regulation essential for adrenal function [26-28]. Primary adrenal insufficiency, commonly known as Addison's disease, typically emerges from autoimmune mechanisms where the body's immune system targets the adrenal glands, leading to hormonal deficits. Other causative factors may include infections, genetic anomalies, or surgical excision of the adrenal glands. Conversely, secondary adrenal insufficiency arises when the pituitary gland falters in producing adequate levels of adrenocorticotropic Hormone (ACTH), consequently impairing cortisol synthesis. This condition can arise from prolonged steroid usage, pituitary tumors, or radiation-induced damage to the pituitary [26,27]. Symptoms of adrenal insufficiency exhibit variation but frequently entail weakness, fatigue, dizziness, weight loss, skin darkening, dehydration, and low blood pressure. Left untreated, the condition can escalate to severe complications like renal failure, shock, and even coma. Diagnosis involves blood and urine examinations to gauge hormone levels, along with imaging studies such as X-rays or MRI scans to pinpoint the underlying cause. Treatment typically revolves around hormone replacement therapy to rectify deficiencies and effectively manage symptoms [26,27].

Hypopituitarism

Specific biomarkers for endocrine disorders, particularly hypopituitarism, encompass a range of indicators crucial for precise diagnosis and effective management. In the context of hypopituitarism, notable biomarkers include TSH, which governs the production of thyroid hormones T3 and T4. Assessing TSH levels aids in evaluating thyroid function status and gauging the response to treatment. Additionally, biomarkers such as cortisol and dehydroepiandrosterone-sulfate (DHEA-S) levels are pivotal for assessing adrenal function. They assist in identifying conditions like Cushing's syndrome or Addison's disease of the adrenal cortex [29]. Furthermore, within the realm of hypopituitarism, growth hormone (GH) deficiency can be assessed through biomarkers like insulin-like growth factor-1 (IGF-1). Insulin-like growth factor-1 plays a critical role in evaluating GH status and directing treatment approaches. Low levels of IGF-1, coupled with inadequate GH secretion upon stimulation testing, signify GH deficiency. These biomarkers are instrumental in diagnosing endocrine disorders like hypopituitarism [29].

Hyperglycemia and Diabetes

Specific biomarkers for endocrine disorders related to hyperglycemia and diabetes encompass a variety of indicators crucial for screening and managing the condition. These include hemoglobin A1c (HbA1c), oral glucose tolerance tests, fasting blood glucose, fructosamine, 1,5-anhydroglucitol, and microalbuminuria [29]. In the domain of thyroid disorders, significant biomarkers include TSH, thyroid peroxidase, thyroglobulin antibodies, and calcitonin [29]. Biomarkers for adrenal function evaluation encompass cortisol, DHEA-S, aldosterone, renin, and metanephrines. These biomarkers aid in testing for conditions such as Cushing’s syndrome or Addison’s disease [29]. Reproductive health monitoring involves biomarkers such as follicle-stimulating hormone (FSH), luteinizing hormone (LH), estrogen, testosterone, progesterone, hCG, inhibin B, and anti-müllerian hormone (AMH), which track ovarian/testicular function and fertility [29]. For neuroendocrine tumors, essential biomarkers include prolactin, chromogranin A, serotonin, gastrin, and insulin-like growth factor 1 [29]. Additionally, adiponectin (ADP) serves as a significant biomarker for obesity and endocrine disruption [30].

Clinical applications and utility

Biomarker-Guided Therapeutic Interventions

The utilization of biomarker-guided therapeutic interventions has garnered increasing recognition as a pivotal component in disease management. These interventions entail leveraging biomarkers, which serve as biological indicators of disease progression or response to treatment, to inform treatment decisions and optimize patient outcomes. Incorporating biomarkers into therapeutic strategies has demonstrated efficacy in enhancing the safety and effectiveness of treatments, particularly in conditions where traditional diagnostic and monitoring methods are limited or unreliable [31-34]. In the realm of heart failure, for instance, the Guiding Evidence-Based Therapy Using Biomarker-Intensified Treatment in Heart Failure (GUIDE-IT) study sought to assess the safety and efficacy of a biomarker-guided treatment approach. This study utilized natriuretic peptide levels to inform adjustments to therapy, with the objective of attaining and sustaining a target level. The implementation of this approach proved beneficial in improving outcomes for patients with heart failure [31].

Likewise, in the management of acute kidney injury (AKI), biomarkers like tissue inhibitors of metalloproteinases (TIMP-2)and insulin growth factor binding protein 7 (IGFBP7) have been employed to guide the implementation of an AKI care bundle. This biomarker-guided approach has demonstrated efficacy in mitigating the incidence and severity of AKI in high-risk patients undergoing major surgery [32]. In the context of Alzheimer's disease, biomarkers are being investigated as potential tools for developing targeted therapies. For example, the phosphatidylinositol 3-kinase (PIK3)/protein kinase (AKT)/glycogen synthase kinase-3β (GSK-3β) signaling pathway has been identified as a promising therapeutic target. Biomarkers such as phospho-GSK-3β (Ser9) are being developed to evaluate the efficacy of lithium treatment in specific patient subpopulations [33].

Monitoring Disease Progression and Treatment Response

Biomarkers play a pivotal role in overseeing the progression of disease and gauging treatment efficacy in both endocrine and metabolic disorders. These biomarkers encompass a diverse array of indicators, ranging from hormone secretion levels and biochemical markers to imaging diagnostics, genetic analyses, patient history, and symptoms [35, 36]. Monitoring biomarkers are systematically evaluated over time to track disease progression, the emergence of new effects, the exacerbation of pre-existing abnormalities, or alterations in disease severity [36]. They also serve to assess the response of a disease or condition to treatment, whether favorable or unfavorable [36]. Within the domain of endocrine disorders, biomarkers like IGF-1 have emerged as crucial indicators for evaluating growth hormone status and guiding treatment strategies, particularly in conditions such as growth hormone deficiency [35]. Moreover, ongoing research endeavors are dedicated to identifying novel biomarkers for the diagnosis and monitoring of thyroid diseases [35]. Circulating biomarkers, such as circulating tumor DNA (ctDNA) and protein biomarkers, are under scrutiny for their potential to enhance therapy monitoring and early detection of disease progression in lung cancer patients [35]. Studies have demonstrated that the median difference in the concentration of ctDNA, cancer antigen 125 (CA125), and Cyfra21-1 was markedly lower in patients exhibiting partial response compared to those with progressive disease [35].

Predicting Outcomes and Prognosis in Critical Illness

Predicting outcomes and prognosis in critical illness poses a multifaceted challenge, involving various factors and methodologies. Although statistical models like the Acute Physiology and Chronic Health Evaluation (APACHE) II score offer valuable predictions, they exhibit limitations when applied to individual patients [37, 38]. While physicians' clinical judgment has demonstrated superiority over APACHE-II in outcome prediction, it still needs to be improved [38]. There is a pressing need for novel early outcome predictors to inform patient and family expectations and decision-making [39]. These predictors should not only anticipate the risk of mortality but also consider disability, enabling the honoring of patient wishes and realistic assessments of functional outcomes [39]. Admission skeletal muscle quality and quantity may serve as pivotal factors in this discourse, with ongoing assessment through lean body mass ultrasound and other modalities deemed essential for continued prognosis discussions [39]. Prediction models aiming to forecast disability and quality of life post-critical illness have been devised to facilitate shared decisions regarding ICU admission, levels of organ support, care duration, and discharge planning [40]. However, many prediction models remain confined to development and need more proven generalizability to alternative settings [40]. Enhancing the clinical applicability of prediction models necessitates external validation through additional datasets [40]. Barriers to implementation include the requirement for real-time data collection and addressing missing predictors through imputation [40]. Transparent reporting of model equations and software compatibility with hospital systems are also pivotal considerations [40].

Emerging trends and future directions

Novel Biomarkers Under Investigation

Extracellular vesicles (EVs) and exosomes have garnered considerable interest in biomedical research, particularly urinary EVs, due to their non-invasive isolation method and diverse cargo composition, including proteins, lipids, and nucleic acids reflective of their originating cells. Recent investigations have identified potential EV-based biomarkers for endocrine hypertension, primary aldosteronism, and renal diseases, showcasing their potential diagnostic and prognostic utility [41]. Neutrophil gelatinase-associated lipocalin (NGAL) has emerged as a promising biomarker and immunomodulator in endocrine hypertension. Ongoing research actively explores its regulatory role and potential mechanisms, offering insights into its applicability as a diagnostic and therapeutic target [41]. Serum diiodotyrosine (DIT) and monoiodotyrosine (MIT) levels, measured through liquid chromatography-tandem mass spectrometry, show promise in differentiating destructive thyroiditis from Graves' disease. Notably, serum DIT levels hold potential as a novel diagnostic biomarker for thyroid disorders, presenting an avenue for enhancing diagnostic accuracy and treatment decisions [42]. Genomic tests like Prosigna play a pivotal role in guiding adjuvant endocrine and chemotherapy decisions for early-stage breast cancer patients. These assays assess the risk of distant relapse based on the expression of specific biomarkers, such as the risk of recurrence (ROR) score, facilitating personalized treatment strategies [43]. Analyzing the transcriptome of urinary EVs, particularly EV-derived mRNA, holds promise for advancing our understanding of endocrine hypertension pathophysiology. This approach may lead to the development of more specific diagnostic and prognostic strategies, contributing to improved patient management and outcomes [41]. These novel biomarkers, particularly EVs and their cargo represent a promising avenue for evaluating the health status of the cardiovascular and renal systems. They hold the potential for diagnosis, prognosis, and treatment optimization across various endocrine disorders, highlighting their versatility and clinical significance [41].

Technological Advancements Enhancing Biomarker Detection

Emerging technologies have ushered in the era of multiplexed detection platforms, allowing for the simultaneous detection of multiple biomarkers. These platforms offer a comprehensive analysis of disease profiles and facilitate personalized treatment strategies by enhancing efficiency and reducing diagnosis time [44]. Cutting-edge ultra-sensitivity detection techniques, such as ultrasensitive immunoassays and digital enzyme-linked immunosorbent assay (ELISA) technologies like Simoa®, have transformed biomarker detection. These techniques enhance early disease detection and improve diagnostic accuracy by enabling the identification of biomarkers at deficient concentrations [45]. Integrating imaging techniques like positron emission tomography (PET) with biomarker detection presents a multidisciplinary approach to improving diagnostics in neuropathology. Novel imaging biomarkers, such as PET imaging for Alzheimer's disease, provide valuable insights into disease progression and assist in early detection and treatment decisions [46]. Advancements in biofluid-based biomarker detection methods, including saliva and urine analysis, have paved the way for non-invasive diagnostics in neurological disorders. Biofluid-based biomarker detection offers easily accessible sources of diagnostic information, facilitating early disease detection and monitoring [46]. Integrating omics technologies, particularly proteomics with mass spectrometry, has revolutionized biomarker identification in various pathologies. These technologies provide a deeper understanding of disease mechanisms and drive the discovery of novel biomarkers for enhanced diagnostic accuracy [46].

Case studies and clinical examples

Illustrative Cases Demonstrating the Utility of Biomarkers in Diagnosis and Management

Insulin-like growth factor-I has emerged as the standard biomarker for evaluating growth hormone (GH) status. In diagnosing and managing GH deficiency, IGF-1 contributes to most clinical and surrogate endpoints for patient diagnosis, treatment, and monitoring. It proves to be a more sensitive indicator of changes in GH status compared to IGF-binding protein 3 (IGFBP-3), which was previously employed as a biomarker in children under eight years old [43]. Predictive biomarkers are crucial in identifying patients at heightened risk of developing immune-related endocrine dysfunctions, enabling proactive monitoring and effective management. For instance, in a study involving 45 patients with advanced malignant melanoma treated with anti-PD-1 antibodies, a baseline absolute eosinophilic count (AEC) exceeding 240/μL correlated with a heightened incidence of endocrine adverse events (sensitivity = 87.5%, specificity = 50%). Moreover, a relative eosinophilic count (REC) surpassing 3.2% at one month during treatment proved a valuable biomarker for predicting endocrine irAEs [47].

Current research endeavors are concentrated on identifying novel biomarkers for diagnosing and monitoring thyroid diseases. These fresh biomarkers hold promising prospects for distinguishing between benign and malignant thyroid neoplasms and differentiating between various benign thyroid disorders presenting with similar clinical manifestations [48]. In the domain of breast cancer, biomarker assays play a pivotal role in guiding adjuvant endocrine and chemotherapy decisions for early-stage patients. Genomic tests like Prosigna have proven instrumental in assessing the risk of distant relapse based on the expression of specific biomarkers, such as the ROR score, thereby facilitating personalized treatment approaches [43]. These instances underscore the diverse applications of biomarkers in endocrinology, encompassing the assessment of GH status, prediction of immune-related adverse events, differentiation of thyroid disorders, and guidance of breast cancer treatment decisions. Using validated biomarkers has significantly enhanced the diagnosis, management, and personalization of care for endocrine-disorder patients.

Real-World Applications and Implications for Patient Care

Digital biomarkers radically impact patient care and outcomes by facilitating personalized remote care, empowering individuals to manage their health actively, and enabling healthcare providers to deliver tailored treatments. This personalized approach enhances patient engagement and adherence to treatment plans, ultimately leading to improved health outcomes [49]. Healthcare providers leverage insights from digital biomarkers to make informed treatment decisions, effectively managing high-risk, high-cost patients, predicting readmissions, and selecting interventions tailored to individual patient needs. This targeted approach improves treatment efficacy and patient satisfaction while reducing healthcare costs associated with unnecessary treatments and hospitalizations [49]. Patient stratification driven by biomarkers optimizes resource allocation in healthcare systems by tailoring treatments to individual patients' needs. This approach minimizes waste, avoids unnecessary interventions, and saves costs, contributing to a more efficient and sustainable healthcare system [50]. Biomarkers have become indispensable tools in drug development, allowing pharmaceutical companies to identify patient populations most likely to benefit from new drugs. Drug development timelines are expedited by targeting specific patient groups, and the likelihood of successful clinical outcomes is increased, driving innovation in medical therapeutics [50]. Digital biomarkers collected outside traditional healthcare settings provide valuable insights into patients' day-to-day health status between physician visits. This real-time data enables precise, patient-specific medical decision-making and facilitates remote monitoring, enhancing patient care and management [49]. Incorporating biomarkers into patient stratification represents a paradigm shift towards personalized and precise medicine. By optimizing treatment efficacy, minimizing adverse effects, and accelerating drug development, biomarker-driven patient stratification redefines healthcare towards a more individualized approach, promoting better patient outcomes and overall health [50].

## Conclusions

In conclusion, this review underscores the pivotal role of biomarkers in the early detection of endocrine disorders amidst critical illness. Through a comprehensive examination of various biomarkers spanning thyroid dysfunction, adrenal insufficiency, and beyond, this study elucidates their significance in diagnosing and monitoring patients within ICUs. By providing insights into the underlying pathophysiology of endocrine dysregulation, biomarkers enable clinicians to swiftly initiate tailored interventions, thereby improving patient outcomes and reducing the incidence of complications. Moreover, integrating biomarkers into routine clinical algorithms holds promise for streamlining diagnostic processes and optimizing therapeutic decision-making in critical care settings. Future research endeavors should focus on validating the clinical utility of emerging biomarkers, leveraging technological advancements to enhance detection sensitivity, and conducting large-scale prospective studies to assess their impact on long-term patient outcomes. Through collaborative interdisciplinary efforts, the potential of biomarkers in early endocrine disorder detection in critical illness can be fully realized, ushering in a new era of precision medicine in critical care endocrinology.
